# Supplementary material for: Defying Death: A Multi-Omics Approach to Understanding Desiccation Tolerance and Senescence in Eragrostis nindensis
Source: Plants (Basel). 2025 Nov 2;14(21):3360. doi: 10.3390/plants14213360 (PMC12610262; doi:10.3390/plants14213360)
Supplement: Supplementary file 1 [file plants-14-03360-s001.zip › Supplementary Figures S1-7.pdf]

Supplementary Figures

Madden *et al.* (2025) Defying death: a multi-omics approach to understanding desiccation tolerance and senescence in *Eragrostis nindensis*. *Plants* 3360. https://doi.org/10.3390/plants14213360

Video S1. Time-lapse of desiccation and recovery in *Eragrostis nindensis*. Visual sequence showing drying and rehydration of the resurrection plant, highlighting recovery in desiccation-tolerant tissues and irreversible damage in senescent tissues.

https://www.youtube.com/watch?v=gV6_CJXM0sw

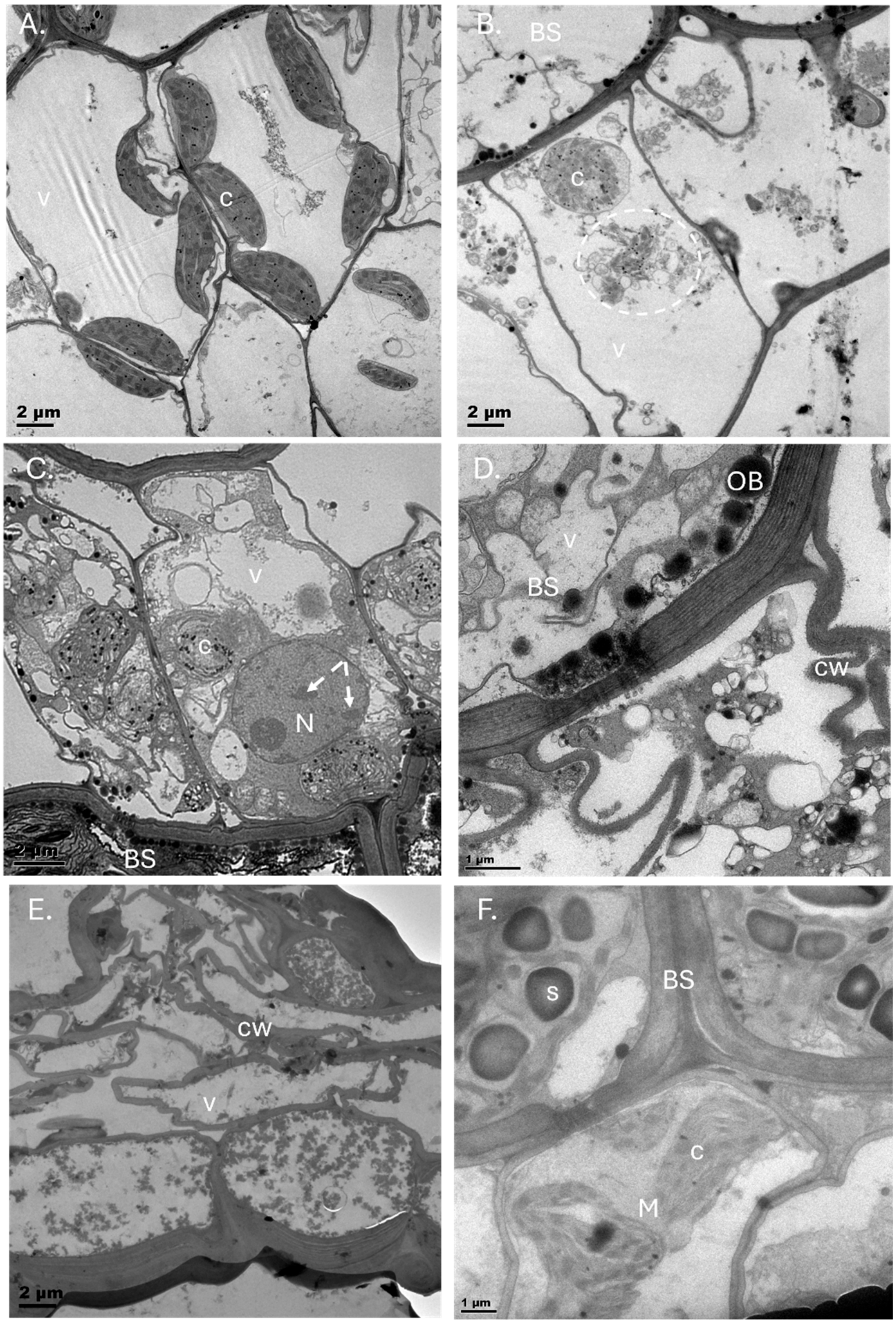

Figure S1: Ultrastructural changes in mesophyll cells of NST of *Eragrostis nindensis* during dehydration and rehydration. TEM images show cellular changes across RWC (%) and after 12 h rehydration. (A) At full hydration (100% RWC), chloroplasts (c) are aligned along the cell wall. (B) At 60% RWC, chloroplasts swell and plastoglobuli appear. (C) At 40% RWC, vacuoles (v) fragment, thylakoids disassemble, and the nucleus (N) displays nucleolus-like bodies (dashed arrows). (D) At 25% RWC, chloroplasts are no longer distinguishable and cell walls (cw) are folded. (E) In the desiccated state (<10% RWC), cells are compressed but membranes remain intact. (F) After 12 h rehydration, mesophyll cells (M) regain full volume and thylakoids are reassembled. BS = bundle sheath cell.

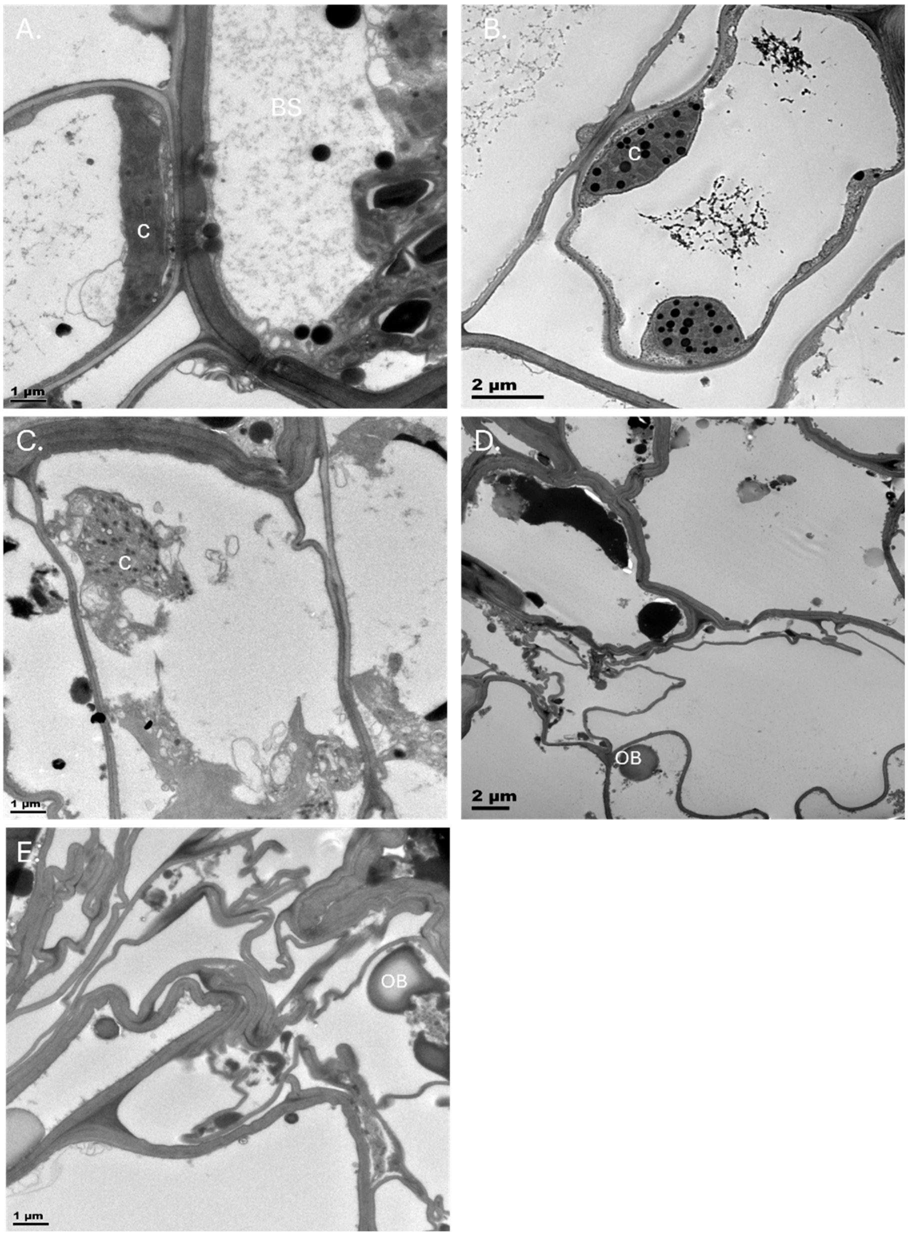

Figure S2. Ultrastructural changes in mesophyll cells of ST of *Eragrostis nindensis* during dehydration and rehydration. TEM images show changes across RWC (%) and after 12 h rehydration. (**A**) At 100% RWC, chloroplasts (c) are fully formed and aligned along the cell wall. (**B**) At 60% RWC, the plasmalemma retracts and enlarged plastoglobuli appear. (**C**) At 25% RWC, cellular disorganisation and plasmalemma rupture (double arrow) are evident. (**D**) In the desiccated state (<10% RWC), only osmophilic bodies (OBs) remain visible and cell walls (cw) are highly folded. (**E**) After 12 h rehydration, organelles are no longer recognisable, and cell walls have collapsed.

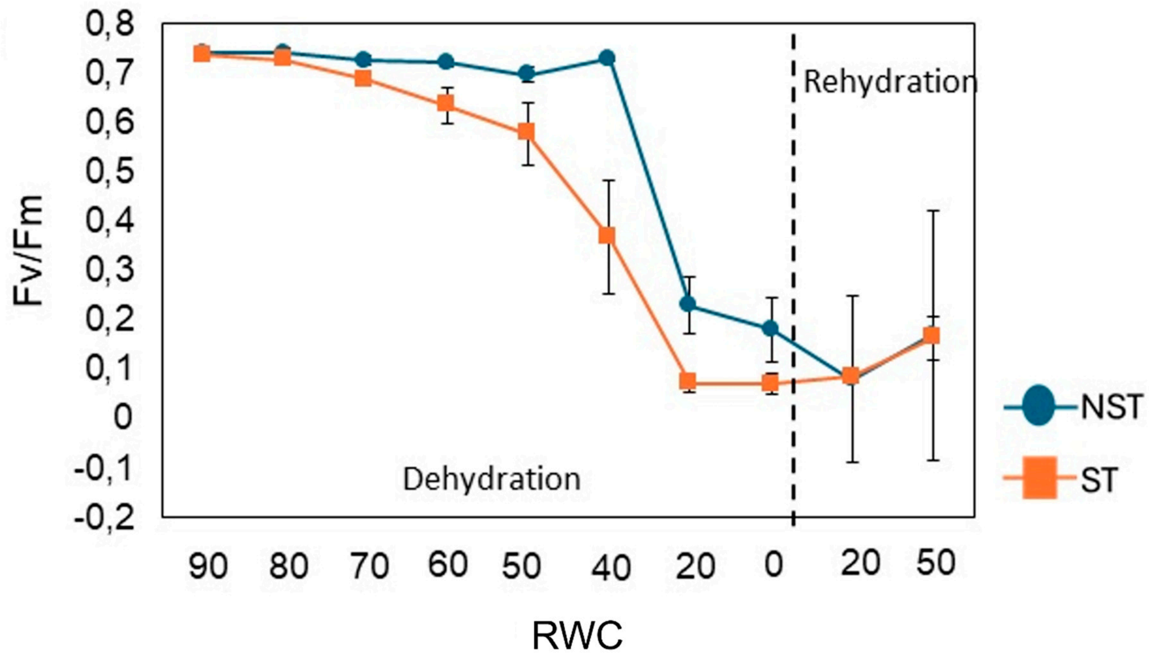

Figure S3. Maximum quantum efficiency of PSII (Fv/Fm) of desiccation tolerant, non-senescent tissue (NST, blue) and desiccation sensitive, senescent tissue (ST, orange) of the resurrection plant *Eragrostis nindensis* upon severe water-deficit stress and recovery thereof (represented as relative water contents, RWC, %). The senescent tissue did not rehydrate beyond 20% RWC. Vertical bars represent standard errors.

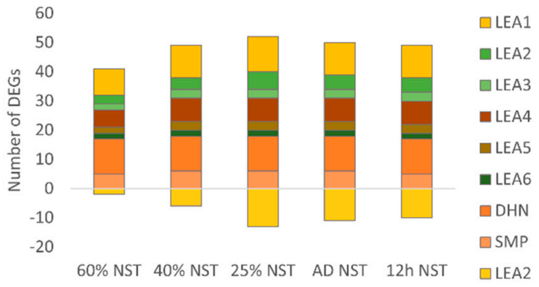

(**a**)

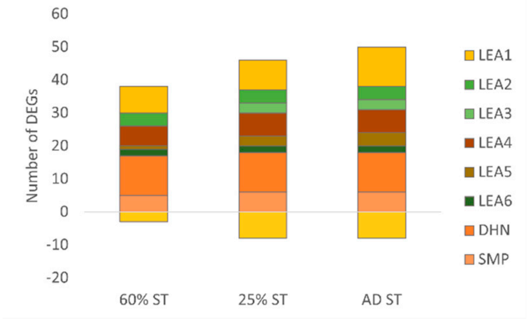

(b)

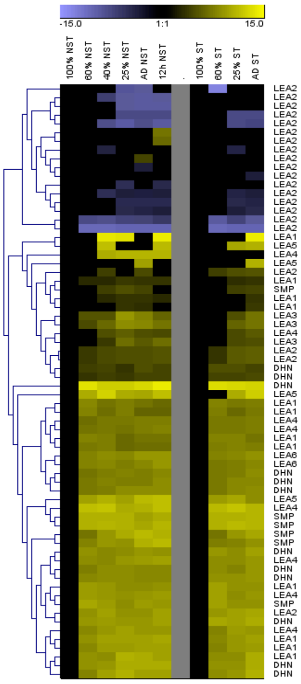

(c)

Figure S4. Expression profiles of LEAs, dehydrins (DHNs), seed maturation proteins (SMPs), and heat-shock proteins (HSPs) during drying and rehydration in *Eragrostis nindensis*. Differential gene expression is shown for NST (**a**) and ST (**b**) across RWC (%) compared to the hydrated control (NST, 100% RWC). Colours indicate LEA family identity. Only statistically significant DEGs ($\log_2$ fold change, FDR < 0.05) are shown. (**c**) Heatmap of DEG trends, with colour scale representing $\log_2$ fold change.

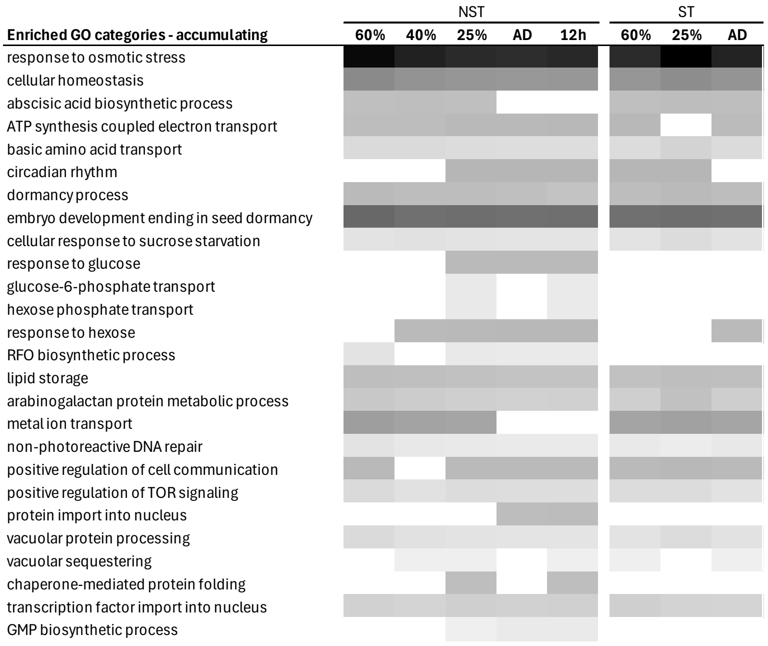

A

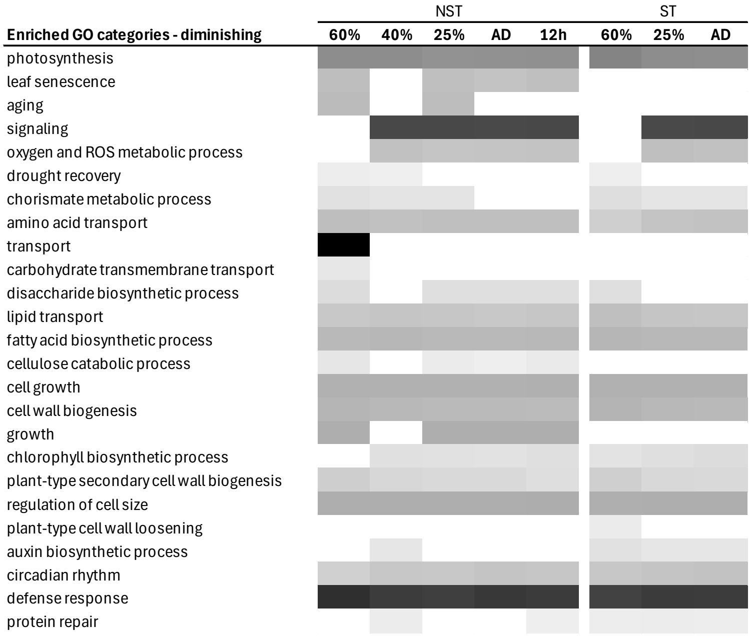

B

Figure S5. Enriched gene ontology (GO) categories during drying and rehydration in *Eragrostis nindensis*. Overrepresented biological process GO terms associated with accumulating (**A**) or diminishing (**B**) transcripts in NST and ST across RWC (%) and after 12 h rehydration. GO terms were identified using BiNGO and summarised with REViGO. Categories shown have q-values < 0.05 (FDR-corrected) and a $\log_2$ fold change > 2 or < –2. Colour intensity reflects the ratio of observed to expected genes, with darker shades indicating greater enrichment.

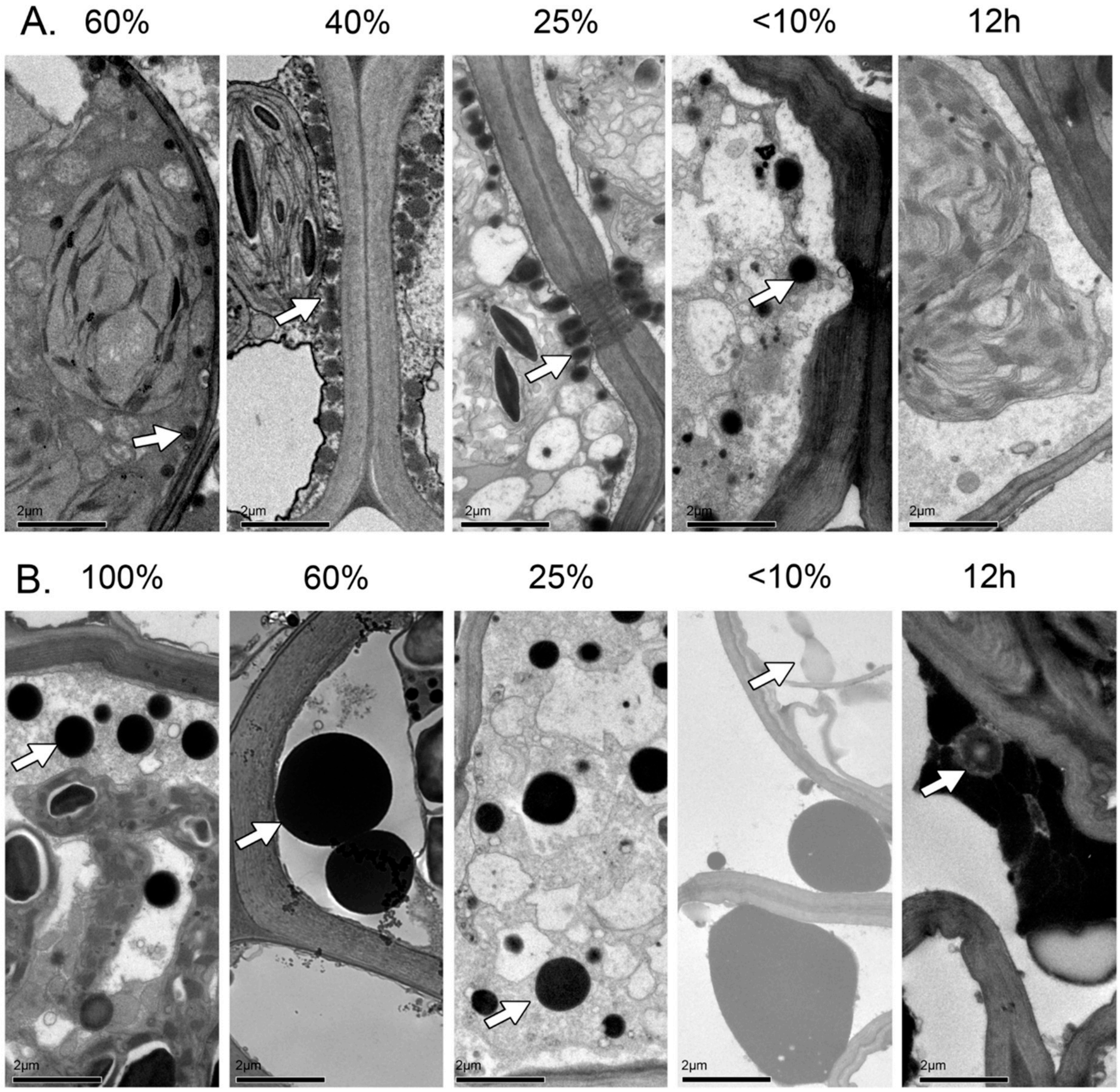

Figure S6. Lipid droplet dynamics differ between NST and ST during drying and rehydration in *Eragrostis nindensis*. TEM reveals LD (white arrows) accumulation in bundle sheath cells. (**A**) In NST, small, regularly sized LDs aligned along the plasmalemma appeared at 60% RWC, increased at 40% RWC, peaked at 25% RWC, diminished in the desiccated state (<10% RWC), and disappeared after 12 h rehydration. (**B**) In ST, osmophilic bodies (OBs) were present at 100% RWC and accumulated irregularly from 60% RWC onward. OBs remained abundant through desiccation and persisted after rehydration, when cells were collapsed and lipid-containing debris indicated cell death.

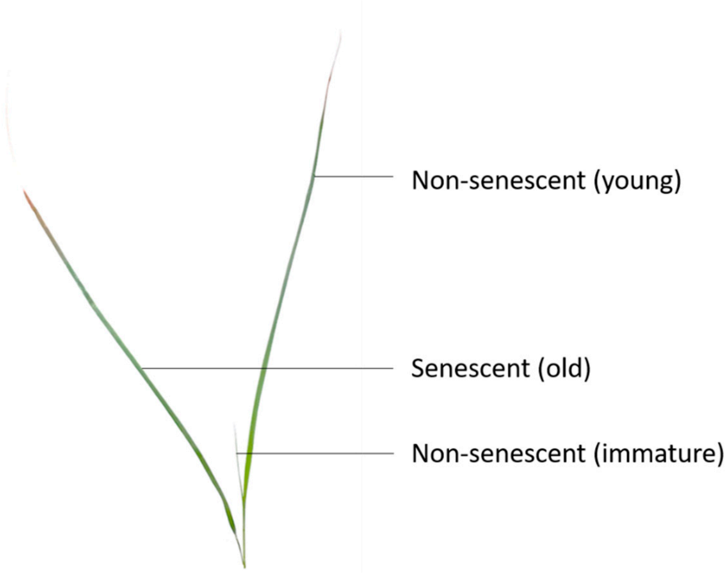

Figure S7. Leaf types sampled for physiological and molecular analyses in *Eragrostis nindensis*. Tiller showing younger, desiccation-tolerant NST leaves positioned closer to the sheath, and older, desiccation-sensitive ST leaves extending outward. Only NST recovers after rehydration, while ST fails to survive desiccation.
